# Cooperative Breeding as a Likely Early Catalyst of Human Evolution

**DOI:** 10.1002/evan.70016

**Published:** 2025-08-28

**Authors:** Judith M. Burkart, Paola Cerrito, Giancarlo Natalucci, Carel P. van Schaik

**Affiliations:** ^1^ Department of Evolutionary Anthropology University of Zurich Zurich Switzerland; ^2^ Center for the Interdisciplinary Study of Language Evolution (ISLE) University of Zurich Zurich Switzerland; ^3^ Department of Neonatology, Family Larsson‐Rosenquist Center for Neurodevelopment, Growth and Nutrition of the Newborn University of Zurich and University Hospital Zurich Zurich Switzerland

**Keywords:** altercentrism, bipedality, brain size evolution, cooperative breeding, neurodevelopment, secondary altriciality

## Abstract

Unlike any other great ape, humans give birth to large, secondarily altricial babies, show precocial social development, have bigger brains that require a long maturation period, and engage in cooperative breeding (CB). These traits, which characterize the human adaptive complex, are intricately linked and must have mutually reinforced each other over evolutionary time. Here, we use recent evidence from paleontology, developmental psychology, and pediatrics, complemented with comparative analyses, to ask what may have triggered this coevolutionary feedback loop: bipedality, direct selection on altriciality, a higher‐quality diet, or cooperative breeding. An early adoption of extensive allomaternal care during human evolution, that is, the CB‐first model, best accommodates the available data. In particular, CB was a catalyst enabling further increases in brain size, because even though larger brains slow down life history and neurodevelopment and thus lead to a demographic dilemma, CB enabled the necessary increase in birth rates.

## 1 Introduction

Despite many similarities, several traits clearly set humans apart from other great apes. Here we focus on the following traits that form an intricately linked nexus of mutually reinforcing, coevolutionary connections between giving birth to large, fat, and helpless newborns [1, 2, 3], having much larger brains associated with delayed maturation and longer adult lifespan [4, 5], and relying heavily on cooperation for subsistence and rearing offspring [6, 7, 8]. While their links are widely recognized, what was the trigger that initiated these evolutionary feedback loops remains debated.

Humans are born helpless. Later birth is most likely impossible because of the narrow birth canal shaped by bipedality and constraints on maternal energetics. Since our ancestors were already bipedal when rapid pulses in brain size increase occurred [9], there was an upper limit for when babies could be born [10, 11, 12, 13, 14]. In addition, in modern humans, the mother's own metabolism is unable to transform enough energy to support any further intrauterine (brain) growth [15, 16]. Both factors clearly prevent human infants from being born later (see also [17]).

Human babies are often referred to as secondarily altricial, relative to other primates who tend to be more precocial, including the nonhuman great apes. Altriciality is defined as a developmental state at birth in which the eyes are still closed, fur or feathers are still absent, and the abilities to thermoregulate and move independently are very limited [18]. In practice, the altriciality‐precociality dichotomy is more of a spectrum. Human babies are born with their eyes open but unable to locomote. Some scientists (especially neurobiologists) therefore use the percentage of adult brain mass present at birth as a proxy for the degree of altriciality. According to this measure, human infants who are born with only about 30% of adult brain mass are more altricial compared with chimpanzees, who are born with about 40% [3, 13, 18]. Most other primates are precocial, with their eyes open and able to move right after birth. Accordingly, their neonate brains are relatively larger than in humans, ranging from around 40% to 70% of adult weight [19, 20, 21]. In strongly altricial mammals, such as rodents or carnivores, these percentages are far lower, ranging from less than 5% to around 30% of the adult brain [22]. It is important to note that the percentage of adult brain mass present at birth is but one of many metrics to consider in defining the state of maturity at birth (see also Section 2.2.1). Overall, relative to other primates, humans are somewhat more altricial (e.g., unable to locomote), and are therefore considered secondarily altricial.

Humans also stand out among apes by having larger brains. Larger brains develop more slowly than smaller ones [5, 23, 24, 25], which, in addition to the earlier timing of birth, increases the immaturity and vulnerability of human newborns [19]. To care for these helpless infants, mothers must rely on systematic allomaternal care, which qualifies us as cooperative breeders [7, 26, 27, 28, 29]. However, hypercooperation characterizes not only our reproduction but our entire subsistence ecology, including cooperative foraging [30, 31, 32, 33, 34, 35]. Across both primates [36] and birds [37], these two traits are tightly linked: general cooperativeness (i.e., proactive prosociality, the key ingredient of human hypercooperation: [38]) increases as species rely more on allomaternal care and cooperative breeding, and humans perfectly fit this relationship [36]. The reach of human cooperation is further shaped by our larger and more powerful brains, allowing us to coordinate future action plans based on language.

### 1.1 What Triggered This Evolutionary Nexus?

**Figure 1:**
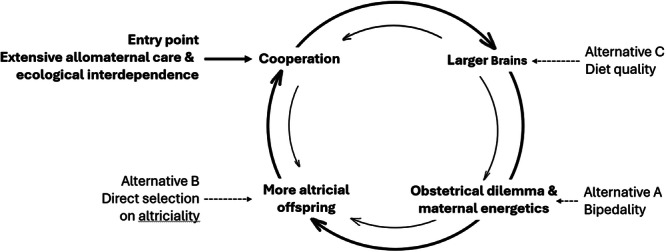
Human cooperation, large brains, morphological and energetic limits to gestation, and altriciality form a tightly interconnected, coevolutionary nexus. The horizontal arrows indicate potential entry points or triggers of this nexus, the one considered most plausible here being extensive allomaternal care.

This general description of the s*tatus quo* of human helplessness at birth and associated traits in modern humans is hardly controversial. In fact, they characterize the human adaptive complex [34] and over evolutionary time, these traits must have mutually reinforced each other in coevolutionary feedback loops. There is thus a nexus of traits that are intricately linked to each other in various ways (Figure 1). For instance, once help in raising offspring was established, it became possible for neonates to become a bit more altricial and for mothers to rely a bit more on others. Likewise, once larger brains and ensuing stronger cognition were established, the reach of what can be achieved via cooperation increased. These linkages make it difficult to identify the causality of how and when the different elements emerged. It is therefore more promising to ask what the most likely entry point into this coevolutionary spiral may have been. The purpose of this paper is to evaluate different scenarios that have been put forward by evaluating each possible initial trigger in turn.

## 2 Evaluating the Triggers

### 2.1 Bipedality

Bipedality has been argued to result in secondary altriciality (see also below) as a mere byproduct of the increasingly narrow birth canal (Alternative A in Figure 1; see [14] for a recent review). However, this is unlikely. First, the obstetrical dilemma only became prominent when brains grew larger *after* hominins had become bipedal [13]. Even if, as has recently been argued, gradual obstetric compromises may have become necessary far earlier than previously thought and are also present in some apes, they are not linked to bipedalism [12, 39]. Second, clear signatures of altricial brain development (i.e., persistence of a fetal pattern of brain growth after birth) typical of modern humans evolved millions of years after hominids had become bipedal [40]. This paleontological evidence makes it unlikely that bipedality was the feature that triggered the feedback loop of Figure 1, although it contributed to earlier birth as brain size increased. Likewise, maternal metabolic constraints are unlikely triggers because they only became limiting once brains already had substantially increased [16] and because the maternal maximum sustained metabolic scope is reached during lactation rather than during gestation [15]. The same is true for the work by Rosenberg and Trevathan [41], which convincingly showed that constraints on birth deriving from bipedality necessitated assistance during birth. However, this only happened after brains had become larger. Moreover, other cooperatively breeding primates, such as marmosets, also give birth in the presence of other group members, who often carry the infants right after birth and may eat the placenta. Social support during birth in humans may thus well have been present before the need for assistance at parturition arose.

### 2.2 Direct Selection on Altriciality for Improved Cognition

Another potential entry point (B in Figure 1) may be direct selection on altriciality, that is, on being born earlier per se, to improve adult cognition. For this to be plausible, the benefits of the additional time spent out‐of‐utero would have to outweigh its costs. Moreover, for altriciality to be a valid trigger of the nexus, it should *lead* to cooperation in infant care. We will turn to these three issues of benefits (See 2.2.2) and costs (See 2.2.3) of being born earlier for the infants, and its evolutionary consequences (See 2.2.4) separately in the following sections, but to evaluate them we must first (See 2.2.1) estimate *how much* earlier than expected human babies are born.

#### 2.2.1 How Altricial are Human Babies?

Portmann [3] introduced the concept of the “extrauterines Frühjahr” and thus argued that human babies spend a full year of life as physiologically immature “extragestate fetuses” compared with other apes. More recent approaches yield less extreme estimates of the deviation from the expected gestation duration. First, perhaps the most widely used measure of human secondary altriciality is the percentage of adult brain size present at birth (e.g., [13, 42]). According to this measure, humans are born 7 months “too early” compared with other great apes. Second, if one models the expected duration of human gestation based on comparative data including locomotion substrate (arboreality or terrestriality), activity period (diurnality or nocturnality), female endocranial volume, body mass, and taxonomy (membership to *Hominoidea* or not), this produces an estimated gestation length for modern humans of only 11 months [5], suggesting that extant human infants are born only 2 months “too early.” Finally, other approaches align ages across species based on hundreds of traits (including neurodevelopmental and behavioral landmarks). These comparative analyses show that human newborns overall tend to be similar in maturity state at birth to great apes. The main exception is our protracted locomotor development [43], essentially contributing to infant helplessness. The wide range of estimates warrants caution against relying on one trait (e.g., the percentage of adult brain mass present at birth) as the sole index of neonate maturity. The human gestation length of 9 months is thus somewhat shorter than expected for a hominid of our brain and body size, but how much earlier depends on the traits considered, and early work suggesting an “extrauterines Frühjahr” seems to have overestimated human secondary altriciality.

#### 2.2.2 Benefits From More Inputs for the Developing Brains

Regardless of exactly by how much human infants are born “too early,” their particularly immature, developing brains encounter both more and more diverse stimuli than they would if still in utero. A widely assumed benefit of earlier birth, therefore, is that these environmental inputs would provide human babies with a head‐start for cognitive development, resulting in more powerful adult cognitive functioning (e.g., [44, 45]). Since brain development is contingent on external stimuli [46], and because developing brains are far more plastic and malleable by inputs than mature ones, these early inputs may be particularly influential for adult functioning. For instance, Cusack et al. [45] proposed that infants may use this time to accumulate information to build a foundational model, analogous to when deep neural networks are pretrained in a non‐goal‐directed way that makes them more stable and generalizable later.

It has been proposed that humans possess species‐specific adaptations to optimize early information acquisition during the period characterized by the combination of near‐complete motor helplessness and relatively precocial sensory systems. Our sociocognitive skills develop particularly early and strongly compared with other great apes [47]. Crucially, these skills facilitate social learning by directing the infants' attention and interests to what is important, which is essential for a species that has specialized in cultural learning [44, 48]. Altercentric influences and biases may also be among these supposedly species‐specific human adaptations for early information acquisition (see Box 1).

**Altercentrism—A species‐specific adaptation for information acquisition by altricial human infants?**

A challenge for learning during the earliest period of life is that infants are not yet motorically mature enough to directly interact with their environment, despite relatively well‐developed sensory systems. This has recently been addressed by a new perspective on human cognitive development, altercentrism [49, 50]. It integrates accumulating evidence that human babies and toddlers, rather than starting out egocentrically (as, e.g., in the Piagetian tradition), are subject to strong altercentric influences and biases, so that they automatically take others' perspectives and basically see the world through the eyes of these others. Through altercentric influences, the presence and perspective of others can affect how individuals perceive, attend to, process, and even remember objects and situations. When the infant's own and the other's perspectives are in conflict, this altercentric influence can be so strong as to override the own point of view (referred to as altercentric *bias* [50]).

An evolutionary proposal [49] for these altercentric tendencies and biases is that they evolved in the context of human secondary altriciality. Because the infants are motorically too immature to cling to their mothers, the latter would often lay them down close‐by while foraging. In this situation, the mother's and the infant's perspectives are not automatically aligned, in contrast to immature apes riding on their mother's back. An altercentric bias in infants to automatically take and even prioritize the mother's perspective, having a particular interest in her targets of attention and ascribing high value to these targets of attention, would be particularly beneficial to acquire relevant information in the secondarily altricial immature human babies.

However, several lines of evidence question whether altercentrism is indeed a species‐specific adaptation for early information acquisition in secondarily altricial human babies. First, it is questionable whether physical separation from any caregiver indeed occurred frequently and long enough during human evolution [51, 52, 53, 54]. Second, it is not restricted to the early developmental period that human infants spend ex utero rather than inside (secondary altriciality), but it persists through childhood and is also present in adults [50]. Second and most importantly, the idea that altercentrism is a species‐specific adaptation to human secondary altriciality is inconsistent with comparative data. A comparative perspective on altercentrism [55] reveals it is also prevalent in nonhuman primates and perhaps beyond, as, for example, suggested by the interspecific distribution of co‐representation [56], a typical altercentric effect (see Figure Box 1). Future research will have to test whether altercentric influences may perhaps be particularly strong in human infants compared with other animals.

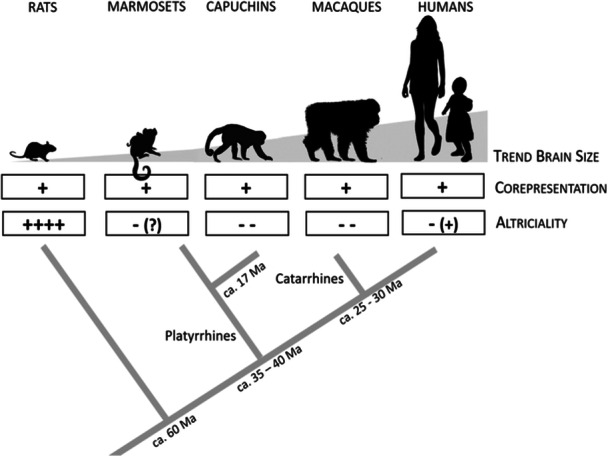

**Figure Box 1:** Co‐representation is not unique to humans. Representing not only the own, but also a cooperation partner's task is a typical alterncentric effect originally described for humans [56, 57]. However, it is not unique to humans but shared with several other primates and rats [58, 59]. Co‐representation is also not related to altriciality or brain size, suggesting it is universal at least in primates, but perhaps also in other social mammals.

Altercentrism, while likely beneficial for information acquisition, thus appears unlikely to be a species‐specific adaptation to human secondary altriciality. In fact, its presence in several nonhuman species has potentially far‐reaching consequences. It provides a novel perspective not only on human development but also on cognitive evolution, by upending the traditional idea that cognition starts out as a private, egocentric endeavor [55].

The strong and early emerging sociocognitive abilities, including altercentric influences and biases, are clearly important during human ontogeny. However, it is less obvious that they are particularly important during the first months after birth (the few months that human infants spend ex utero rather than inside) rather than later during infancy and childhood. Moreover, these cognitive abilities not only facilitate information acquisition, but also help immatures to tackle the specific demands of being cared for not only by mothers (who tend to care for infants conditionally depending on the perceived availability of help among cooperatively breeding primates, in both humans [60] and marmosets [61]), but also by others. These others are not always available, and not always equally motivated, resulting in social selection pressures to develop strong early sociocognitive skills to solicit care from mothers and others [62].

#### 2.2.3 The More Inputs the Better? Lessons From Preemies

In contrast to the prediction that particularly early inputs in the developing human infant brain are beneficial for adult cognitive function, recent evidence suggests that more is not always better. Rather, getting the right input at the right time may be more important than getting as much as possible as early as possible. For instance, facial expressions are particularly important for species with allomaternal care [63], and particularly dense visual input of facial stimuli is characteristic of early human development [64]. However, too much input too early seems problematic, as suggested by the example of human newborns treated for congenital cataract [65]. The infants subject to this treatment have abnormally high initial retinal acuity relative to typical newborns, but later exhibit *impairments* in configural face analysis, which suggests that the early swamping with visual information was detrimental rather than beneficial [65]. These clinical findings contradict the prevalent idea that more inputs for developing brains are universally beneficial. Such potential costs of altriciality can be further tested by examining prematurely born babies.

Prematurely born infants who survive thanks to medical progress and thrive are a critical test case. If the extra time gained by being born earlier is indeed so precious and beneficial because more inputs can be gathered earlier in neurodevelopment, it should be beneficial to be born even earlier because these extra inputs should contribute to building a particularly powerful cognitive system. Perhaps the bottleneck during evolutionary history was that such more fragile babies were too delicate to handle, even for the most careful mothers and caregivers. Data from neonatal intensive care units shows that in premature babies, even minimal trauma easily leads to brain bleeding with lethal effects in infants that are born too early. However, neonatal‐perinatal medicine (incidentally, an institutionalized form of shared infant care) has found ways to safely handle these babies, and infants born after only 32, 28, and sometimes even 23 weeks of gestation (instead of 40) can nowadays survive and thrive [66].

These thriving preemies are highly informative test cases for evolutionary anthropologists to examine the prediction that an earlier onset of external inputs is advantageous for future cognitive development. However, in strong contrast to this prediction, large cohort studies unambiguously show a spectrum of long‐term developmental adversities, resulting in impairments in cognition [67, 68, 69], language [70], attention, social skills, and in a higher incidence of internalizing symptoms such as anxiety [71] and autism‐spectrum disorder [72].

It could be argued that these impairments are caused by comorbidities of prematurity. First, in addition to biological factors [73, 74, 75], early environmental factors such as exposure to stimuli (light, noise, pain) and deprivation of social contacts during neonatal intensive care also appear associated with altered brain architecture and connectivity and underlie impaired neurodevelopment of preterm‐born children [76, 77, 78]. However, there is increasing evidence that the immature births with the best outcome prognosis are those that are *shielded* from external stimuli (with dimmed light, cribs covered with sheets, sound‐attenuated rooms, and only gentle touching), which is implemented as best practice in neonatal care units [79, 80].

**Figure 2:**
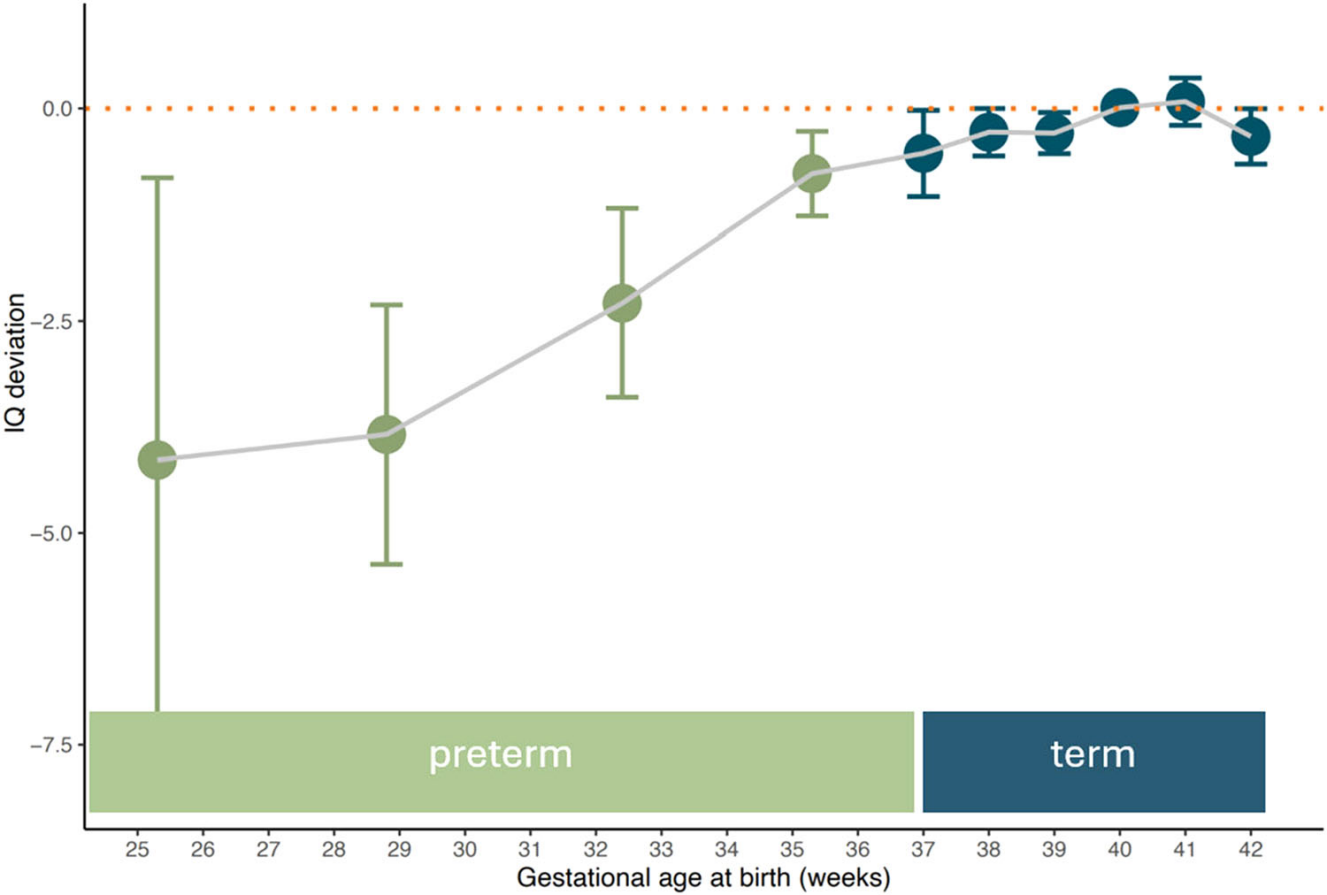
IQ test scores (deviation from norm) at the age of 18 years relative to gestational age at birth, after Husby et al. [68]. Blue indicates gestational age at birth considered *term born*, green refers to *preterm birth*, that is, before 37 weeks of gestation [84].

Second, even if the chances of survival increased during recent decades, preterm born infants are still at risk for a wide range of health problems due to the fragility of their still‐developing bodies at birth, such as brain damage, chronic lung disease, and infections, and these problems are inversely proportional to the prematurity of the exposure to the extra‐uterine environment [81, 82, 83]. A recent population‐based study of 1.2 million Danish children on the consequences of prematurity therefore controlled for morbidity [68]. At the age of 15–16 years, the reduction in standardized school grades (i.e., in written language and mathematics) persisted even after correcting for sociodemographic (i.e., parental education, sex) and medical mediators (i.e., morbidities, hospital admission). Likewise, in a subset of the full sample (227’403 males), IQ scores at the age of 18 years were available. IQ scores decreased with more extreme prematurity, even if controlled for relative birth weight, malformations, parental age, parental educational level, number of older siblings, and shared family factors. Thus, prematurity leads to cognitive deficits rather than improvements, proportionally to the degree of prematurity (Figure 2, after Husby et al. [68]).

To summarize Section 2.2 so far, the cognitive benefits of the human head‐start into life may have been overestimated and its costs neglected. First, as discussed elsewhere [62] the more helpless infants, and thus their mothers, need more help. The necessity for immatures to recruit this help has led to specific selection pressures in the infants that are responsible for the strong and early developing social skills which at the same time optimize information acquisition. However, these pressures are not restricted to the few months of life that humans may be born earlier than expected, but are arguably much stronger later, perhaps peaking around the age of around when the next sibling is born in natural‐fertility nomadic foragers (around 3.3 years [85]). At this moment, the newborn requires all the attention from the mother while the previous child is still very dependent on help and thus needs all the sociocognitive skills to recruit caregivers. Second, a strong argument can be made that early inputs fundamentally shape the growing brain and altercentrism functions to extract even more meaningful information from the environment and thus to optimize the inputs. Nevertheless, altercentrism is also not restricted to, or strongest during, the first few months of life, nor is it unique to humans [55]. Finally, inappropriately early input is clearly detrimental, rather than beneficial for future cognitive performance, as clearly shown by follow‐up studies of prematurely born infants.

A broader neurodevelopmental perspective would also not predict that the earliest environmental inputs are necessarily the most consequential ones, compared with later when higher association cortical fields mature. Developmental neuroscientists distinguish between experience‐expectant and experience‐dependent influences of environmental stimuli on brain development [46]. The former typically involve more basic information processing circuits, and the timing of the input is critical, as evident in the classical example of the influence of postnatal monocular deprivation and on the formation of ocular dominance columns [86, 87]. The experience‐dependent influences are typically more prominent later in development, for example, data showing that growing up with siblings speeds up Theory of Mind development (e.g., [88]), and much less dependent on the exact timing.

In conclusion, although environmental inputs are vital for later cognitive functioning [45, 89, 90, 91], their role during the few months that humans are born earlier than expected may have been overemphasized. Direct selection for secondary altriciality for adult cognitive functioning thus seems unlikely, and thus an unlikely trigger of the coevolutionary nexus in Figure 1.

#### 2.2.4 Allomaternal Care as Consequence of Altriciality?

For the sake of argument, let's nevertheless assume that the cognitive benefits of earlier inputs were strong enough for direct selection on secondary altriciality. To function as the entry point into the feedback loop of Figure 1 (alternative B), altriciality must also have directly resulted in more help with raising offspring. We can use comparative evidence to test whether evolutionarily, altriciality leads to cooperative breeding.

Altriciality and cooperative breeding are linked among bird species. Cooperative breeding is particularly prevalent among birds (about 15%–25% of bird species) because there are more opportunities to help than in mammals (about 2.5%–3%), given that in mammals only mothers can lactate [92] and allonursing tends to be rare [93]. In altricial birds, the opportunities for help and provisioning are larger than in precocial ones, where parents do not provision young [94]. The consistent association between altriciality and cooperative breeding in birds gave rise to the notion that altriciality played a key role in the evolution of cooperative breeding [95].

**Figure 3:**
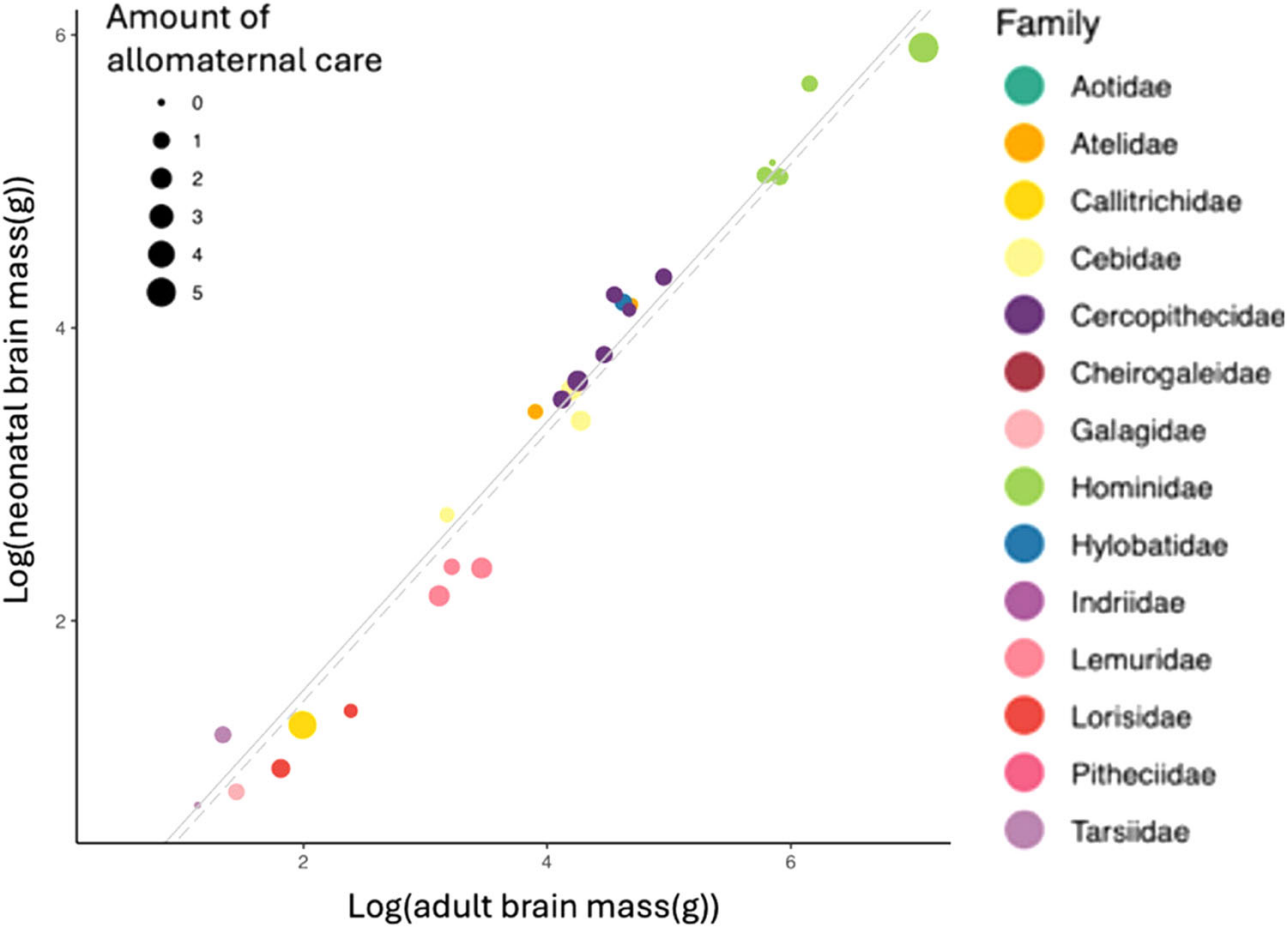
In primates, allomaternal care has a small, but significantly negative effect on % of brain size present at birth. The continuous line represents the PGLS regression without allomaternal care (allomaternal care = 0), while the dotted line represents the PGLS regression with allomaternal care = 5 (which is the maximum value present in our data set). The intercepts of the two lines are small but significantly different (*p* = 0.005), indicating that neonatal brain mass as proportion of adult brain mass is smaller for species with high allomaternal care. PGLS, phylogenetic generalized least squares.

To further investigate whether the link between altriciality and help also holds in the case of human evolution, we zoomed in on primates and analyzed data of 26 primate species across 14 families (neonatal brain mass: [24], adult brain mass: [93]). We used Phylogenetic Generalized Least Squares (PGLS) to assess the relationship between neonatal and adult brain mass while accounting for the nonindependence of the datapoints because of phylogenetic relatedness. The results in Figure 3 show that in primates there is a small, but significant difference in altriciality (measured as proportion of adult brain mass at the time of birth) between cooperative and noncooperative breeders.

But is it likely that the secondary altriciality of humans *preceded* reproductive cooperation, as suggested by alternative B in Figure 1? The link present in both birds and primates is intuitive since more altricial young require more care, and the mere opportunity to engage in allomaternal care is larger in altricial compared with precocial species. Nevertheless, more recent studies in birds suggest that the link between altriciality and cooperative breeding may be weaker than originally thought and cooperative breeding may even have evolved *prior* to the origin of altriciality [96, 97]. Moreover, the absence of allomaternal care in numerous altricial rodents shows that altriciality does not automatically lead to allomaternal care. Finally, in primates, the association in Figure 3 not only has a small effect size but it may also be strongly driven by callitrichid monkeys, who show the highest level of allomaternal care and are the only primates to fully qualify as cooperative breeders [98]. Callitrichids usually give birth to twins, and twinning per se may be responsible for their smaller size at birth, because of spatial and metabolic maternal constraints [15].

Overall, then, the correlation between altriciality and help seems to merely reflect the higher opportunities for help in more altricial species, without altriciality per se strongly driving reproductive cooperation. In any case, an altriciality‐first scenario is implausible for human evolution because it is unlikely that altriciality preceded help. In humans, the presence of highly altricial, fragile offspring that need to be carried and cannot be parked and left behind requires that mothers receive assistance to successfully raise the more needy and helpless infants [7]. If mothers were unable to care for more altricial offspring on their own, starting to give birth to more altricial offspring would have been an evolutionary dead end (cf. [99]. for twinning in callitrichids). More altricial human offspring thus could not evolve *before* help was available.

Taken together, support for an altriciality‐first alternative is empirically and logically weak because the benefits have been over‐ and the costs underestimated, and allomaternal care is more likely a precondition for, rather than a consequence of, human secondary altriciality.

### 2.3 Larger Brains Because of Higher‐Quality Diet

Another entry point could be the evolution of larger brains enabled by the availability of a higher‐quality diet (Figure 1, C), for example, through more meat [100, 101, 102, 103, 104, 105], a shore‐based diet [103], or food processing and cooking [104, 105]. Brains are not only beneficial, but also very energy‐hungry organs, in particular during early ontogeny [106]. A larger brain can only evolve if this energy can be made available, either by redirecting it away from other functions (such as digestion, reproduction, or locomotion) toward the brain, by following heterochronous brain‐body growth patterns [106], by generating a larger energy surplus, or both [100, 107, 108]. These high energy requirements may well explain why human babies are born with a much higher percentage of fat tissue compared with other great apes [107], to buffer them from the nutritional disruption commonly occurring after birth before lactation is established [2]. During gestation, human mothers thus must not only heavily invest in the growing brain of the fetus but also their fat buffers, and once the infants are born must continuously provide enough calories for many years [109] because in contrast to other organs, developing brains cannot be starved temporarily without detrimental effects [110].

Higher‐quality and more energy‐rich food can reasonably provide such additional energy. However, a mere switch in diet that results in higher caloric intake may not be enough to warrant the evolution of larger brains. First, a better‐quality diet, in particular meat, tends to be more difficult to acquire. A woman may well be able to acquire enough energy for herself, provided she can freely move and forage, but because larger brains require earlier birth and thus more helpless babies, efficiently hunting or collecting enough food for herself *plus* the baby is next to impossible. The mother will therefore need help from others, who either take care of the infant while she acquires the food, or who acquire and share it with her and her offspring. Food processing and cooking is perhaps more readily possible with dependent offspring, but it still requires raw food acquired by others who have to be psychologically prepared to systematically share food. Comparative studies show that such a sharing psychology most readily evolves in cooperatively breeding species [36, 111, 112]. Nevertheless, once better‐quality food is available to mothers and their babies through allomaternal care, this further amplifies the opportunities for helping because these foods can be fed to infants by others much earlier than hard to digest plant foods, providing the feedback loop that makes help even more efficient.

Second, and most importantly, a mere switch in diet per se is unlikely to account for the evolution of the large human brains because brains not only imply energetic costs but also life history costs and demographic consequences. In the case of great apes, these costs significantly increase extinction risk [107, 113]. As we will see below, these latter two types of costs, that is, life history costs and demographic costs, had to be buffered by the life‐history consequences of extensive allomaternal care and cooperative breeding. These, therefore, had to be present before, and consequently appear as the most likely entry point in Figure 1.

### 2.4 A Cooperative Breeding‐First Model

Of the three potential entry points, the weight of evidence suggests CB‐first as the most feasible. The early adoption of allomaternal care and ensuing ecological interdependence enabled the evolution of larger brains, which not only convey benefits but also considerable costs, as emphasized by the expensive brain framework [100, 107]. Traditionally, a major focus has been on the costs stemming from the high energetic needs of brain tissue (see above), in particular during ontogeny [106], but bigger brains also have life history costs and the resulting risk of demographic nonviability, and thus extinction (Table 1). To evolve a bigger brain, a species therefore not only needs to be able to pay the increased energetic cost but also to alleviate the additional life history and demographic costs.

**Table 1:** Costs of bigger brains, and how they could be paid during human evolution.

Costs of bigger brains	Hypotheses for how they were paid during human evolution
Energetic costs	More energy because of better diet: e.g., meat^a^, shore‐based diet^b^, processing and cooking^c^ Different energy allocation: less to locomotion (bipedality), gut tissue or reproduction, more to brain^d^ Allomaternal care: more energy from provisioning of immatures by others^e^
Life history costs	Allomaternal care: shortens birth intervals and increases fertility in primates, allowing human mothers to shorten birth intervals *despite* immatures developing more slowly^f^
Demographic costs	Allomaternal care: risk of extinction (the great ape “demographic dilemma” due to slow life history) is attenuated through increased fertility → higher *r*_max_ f

^a^ Bunn [101].

^b^ Cunnane and Crawford [103].

^c^ Wrangham [114].

^d^ Aiello and Wheeler [100]; Isler and van Schaik [107]; Navarrete et al. [108]; Pontzer [115].

^e^ Hrdy [7]; Burkart et al. [26].

^f^ Isler and van Schaik [5, 107].

Allomaternal care contributes to preventing these negative consequences. Comparative evidence shows that generally among primates, bigger brains are associated with a slower life history, indexed by longer gestation, lactation, interbirth intervals, later age at first reproduction and longer life spans and maximum life span [5, 107]. As a consequence, bigger brains are also associated with lower total fertility because the longer life span only partially compensates for the later age at first reproduction and longer interbirth intervals. In other words, as the brains of our ancestors grew larger, their life history slowed down, and their fertility decreased: the great ape “demographic dilemma” [7, 113] worsened. With the values we now have available, one can in fact estimate a proxy for maximum reproductive capacity, namely *r*_max_, the maximal possible population growth rate. Once *r*_max_ falls below a critical threshold, a population is no longer viable and hits the “gray ceiling” [5]: any further brain size increase would lead to extinction. For independently breeding primates, this threshold value of brain size has been estimated at around 665 cm^3^, that is, somewhat larger than in extant great apes (female brain size: between 326 cm^3^ in bonobos and 434 cm^3^ in gorillas) but far less than the size of modern humans (1213 cm^3^). In short, an independently breeding great ape with our brain size and our fertility is demographically impossible.

Our ancestors managed to break through this gray ceiling by greatly reducing interbirth intervals compared with what would be expected for a great ape with our brain size [5], giving rise to our hybrid life history that combines slow growth and long life span with fast reproduction [116, 117]. Comparative evidence shows that across primates, allomaternal care increases fertility by decreasing the duration of lactation [107, 118], which suggests that our ancestors were able to increase their fertility because of their reliance on allomaternal care. Our ancestors thus managed to increase their fertility despite having more slowly developing immatures, because others helped mothers to care for and feed their offspring so that mothers could afford to wean them early and thus become pregnant again. In fact, in contrast to other great apes, human mothers have their next offspring long before the previous one is independent. A strong argument can thus be made that before evolving larger brains, extensive allomaternal care must have been in place.

Across both birds and mammals, increasing fluctuations in environmental conditions and food availability play a crucial role in the evolution of extensive allomaternal care or cooperative breeding [92, 119, 120]. In the mammal pathway to cooperative breeding, the emergence of male care appears as a critical steppingstone (but see [121, 122]). As reviewed in van Schaik [123] and Burkart et al. [92], the earlier forms of the genus *Homo*, around 2 Ma living in arid savannas, faced high seasonal variation in environmental conditions and food availability [124], most likely lived in large mixed sex groups as other terrestrial primates [125], and engaged in communal defense against predators as well as cooperative scavenging and hunting. The latter arose from cooperative defense by hominin males on the open savanna [126], which in turn provided ample opportunities for sharing [30], including of meat, and often between sexes. This sharing system, in turn, paved the way for the mutual interdependence of the sexual division of labor in human foragers [123, 127]. Accordingly, sexual dimorphism in body size was reduced relative to earlier hominins such as *Australopithecus afarensis* [128]. Not only could human immature newborns hardly be reared successfully without extensive allomaternal care, but adult interdependent human foragers likewise could not survive without extensive within‐band sharing. Reproductive [7, 26] and ecological interdependence [30] are thus tightly intertwined in humans (see also [31, 32, 33, 116, 129]). It remains difficult to disentangle which of the two emerged first, but a reproductive interdependence first model again appears more parsimonious. First, a general sharing psychology, including between adults, which is necessary for the implementation of environmental interdependence, easily coevolves together with allomaternal care in primates [36]. Second, it has long been noticed that the proximate mechanisms that support cooperation outside the rearing context piggyback on the evolved infant care system, in particular, the hormonal regulation via oxytocin [130]. Once this infant care system is not only activated in mothers in response to their own offspring but more broadly in all in‐group adults, as is typical for cooperative breeders [131, 132], general in‐group cooperativeness can easily emerge.

Finally, this CB‐first model is also consistent with a recent conclusion from dental evidence in *Homo erectus* [133], strongly suggesting that extensive allomaternal care predated the emergence of the large brains characteristic of modern humans, and that a reduction in age at weaning (which is a hallmark of CB) also predated large brains, as seen in Australopithecines [134].

## 3 Conclusions

The goal of this contribution was to use recent comparative data to explore the evolutionary origin of a nexus of tightly interrelated traits that sets humans apart from other great apes, including larger brains, constraints on extending gestation, secondary altriciality, allomaternal care, and cooperation. Inferring evolutionary causality is notoriously difficult because several of the connections among these traits are likely to form coevolutionary feedback loops. Our approach here was therefore to ask whether we could identify the most plausible entry points that served as the initial trigger for this nexus.

The paleontological record shows that bipedality is an unlikely trigger point because altricial brain development typical of modern humans evolved long after hominids had become bipedal. Direct selection on altriciality, as an alternative trigger, may have occurred because additional time to learn early in life may head‐start human cognitive development. Whereas this period is no doubt important, its role may nevertheless have been overemphasized. First, we find that altercentrism, a supposedly human‐specific cognitive adaptation to this early period of increased helplessness, is present far beyond this initial period, and more importantly, in other primates as well. Second, long‐term data from prematurely born infants show that further extending this period does not provide any additional benefit: their premature brains cannot efficiently use these additional inputs to boost cognitive development. Improved diet quality could have triggered the coevolutionary loop because it clearly played an important role for the evolution of our bigger brains, but a better diet alone cannot explain how the life‐history and demographic costs of the larger brains could have been paid. These latter costs could only be paid by extensive allomaternal care and cooperative breeding, which systematically shorten age at weaning and increase fertility across mammals, including primates.

After evaluating the various triggering scenarios, we conclude that allomaternal care was the most likely necessary *precondition* for the evolution of the nexus. Importantly, this CB‐first model is also consistent with recent conclusions from dental evidence in *H. erectus* [133] and Australopithecines [134], showing that large brains emerged *after* extensive allomaternal care and a reduction in weaning age. Future progress in the use of hard tissue to reconstruct hominin life‐histories will be essential to further corroborate the CB‐first model.

**Figure 4:**
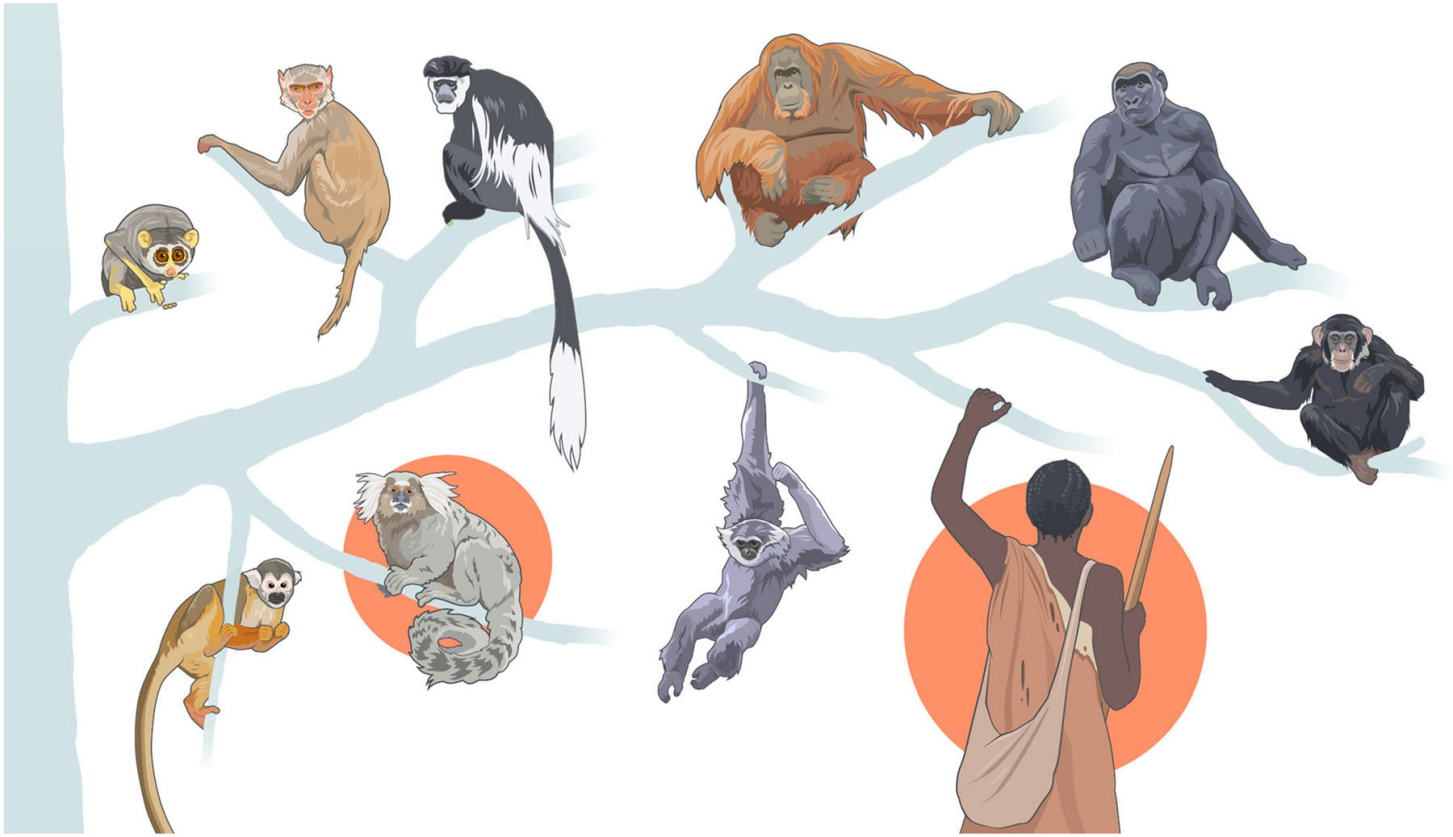
Among primates, only humans and callitrichid monkeys (tamarins and marmosets) qualify as cooperative breeders. According to the cooperative breeding model of human evolution, starting to engage in extensive allomaternal care had cascading effects on a broad range of traits part of the human adaptive complex. Illustration: UZH, ZI, MELS/SIVIC, Mathias Bader.

Among primates, only humans and callitrichid monkeys engage in extensive allomaternal care and cooperative breeding (Figure 4); callitrichids therefore play an important role for understanding human evolution [26, 135]. Both humans and callitrichids are born a bit earlier than expected, but the role of these additional environmental inputs early in life seems moderate. However, by broadening the perspective away from altriciality (i.e., the developmental state at birth) toward entire neurodevelopmental trajectories, Cerrito et al. [136] find systematic differences in when critical life events happen relative to the neurodevelopmental stage in cooperatively versus independently breeding species. In particular, marmoset neurodevelopment extends into adulthood, as in humans, and there are striking similarities in how behavioral milestones linked to cooperative breeding map onto these developmental trajectories. For instance, in both human and callitrichid immatures, the critical change in role from being recipients of help to becoming providers of help occurs when their brains are still differentiating. The implementation of such tasks and associated skills early in life over and over again in a huge and extremely plastic brain must play an important role in shaping the hypercooperative adult human phenotype [91]. This emerging focus on comparative neurodevelopmental timing is a promising way forward to comprehensively capture the consequences of cooperative breeding.

A useful working model to understand human evolution is that of a double legacy [26]. On the one hand, we share many traits with our closest relatives, the other great apes, inherited from the last common ancestor. In particular, from our great ape‐like ancestors we inherited brains that were already very large and slowly developing, and thus had the time to accumulate and integrate large amounts of information during ontogeny. On the other hand, there are traits that have convergently evolved in cooperatively breeding primates and are therefore uniquely present in humans and the cooperatively breeding callitrichid monkeys. These traits are absent in the other great apes who do not systematically engage in allomaternal care and cooperative breeding. Among these traits, we find the life‐history consequences of cooperative breeding, such as the short birth intervals and high fertility despite overall slow development [7]. In humans, they enabled the emergence of even bigger brains, which otherwise would have led to a demographic collapse. The convergently added traits also include higher overall cooperativeness [36], strong sociocognitive skills [62, 137], the timing of key life events during neurodevelopment [136], and conditions favorable for the evolution of human language [135].

## Data Availability

Data sharing is not applicable to this article as no new data were created or analyzed in this study.
